# Analysis of clinicopathologic and imaging features of dual-phenotype hepatocellular carcinoma

**DOI:** 10.1038/s41598-024-53831-5

**Published:** 2024-02-09

**Authors:** Ketuan Huang, Yongfei He, Tianyi Liang, Shutian Mo, Yuan Liao, Qiang Gao, Xiwen Liao, Chuangye Han, Guangzhi Zhu, Tao Peng

**Affiliations:** 1 Department of Hepatobiliary Surgery, The First Affiliated Hospital of Guangxi Medical University, Shuang-Yong Rd. 6, Nanning, 530021 Guangxi Zhuang Autonomous Region People’s Republic of China; 2 Guangxi Key Laboratory of Early Prevention and Treatment for Regional High Frequency Tumor, Nanning, Guangxi Zhuang Autonomous Region People’s Republic of China; 3 Guangxi Key Laboratory of Enhanced Recovery After Surgery for Gastrointestinal Cancer, Nanning, Guangxi Zhuang Autonomous Region People’s Republic of China

**Keywords:** Dual-phenotype hepatocellular carcinoma, CK7, CK19, Prognosis, Cancer, Medical research

## Abstract

Dual-phenotype hepatocellular carcinoma (DPHCC) is a new subtype of hepatocellular carcinoma (HCC). This study aimed to investigate the relationship between the computerized tomography scan (CT) imaging and clinicopathologic features of DPHCC. The CT imaging and clinicopathologic data of 97 HCC cases who underwent radical resection were collected retrospectively. The CT imaging feature was evaluated by the ratio of the average CT value of tumor to liver (TLR) in the plain scan, arterial, portal vein and delayed phases. The association between CT imaging and clinicopathologic features was analyzed using the *t*-test or chi-square test. Univariate and multivariate recurrence-free survival (RFS) analysis and overall survival (OS) were performed. The positive rates of cytokeratin 7 (CK7) and CK19 were 35.1% and 20.6% respectively. The positive rate of CK19 was significantly higher in cases with age < 47 years (*P* = 0.005), tumor diameter > 4 cm (*P* = 0.016) or AFP ≥ 400 ng/ml (*P* = 0.007). The TLR in the portal vein phase was significantly lower in CK19 positive group (*P* = 0.024). The recurrence risk was significantly higher in cases with CK19 positive (HR: 2.17, 95% CI 1.16 to 4.04, *P* = 0.013), tumor diameter > 4 cm (HR: 2.05, 95% CI 1.11 to 3.78, *P* = 0.019), AFP ≥ 400 ng/ml (HR: 2.50, 95% CI 1.37 to 4.54, *P* = 0.002) or CA199 ≥ 37 U/ml (HR: 2.23, 95% CI 1.12 to 4.42, *P* = 0.020). However, imaging features, pathological subtype, CK7 or CK19 expression were not significantly related to HCC OS in the univariate and multivariate analysis (all *P* > 0.05). The expression of CK19 may be associated with the enhancement feature of the portal vein phase CT image, and CK19 positive may suggest a worse RFS.

## Introduction

Primary liver cancer is one of the most common malignant tumors and also one of the main causes of cancer-related deaths in the world^1,2^. In 2012, there were nearly 782,500 new cases and about 745,500 deaths from primary liver cancer worldwide^3^. Hepatocellular carcinoma (HCC) is the predominant type of primary liver cancer, constituting 70–90% of cases^4,5^. The occurrence of HCC is an extremely complex process, and there is currently no clear mechanism that can explain it, but many epidemiological studies have shown that the main risk factors include hepatitis B or C virus chronic infection, long-time alcoholism, and aflatoxin exposure^6–10^. Although the development of integrated treatment approaches based on surgery has greatly improved the clinical prognosis of HCC patients, the postoperative recurrence rate is still high, and the long-term prognosis is still not ideal^11–14^. Currently, some clinical indicators and tumor staging systems have been reported that can predict the prognosis of patients, such as the Barcelona Clinical Liver Cancer Staging (BCLC), serum alpha-fetoprotein (AFP), tumor size, portal vein tumor thrombus (PVTT), and the peripheral blood neutrophil-to-lymphocyte ratio^15–23^. However, the individual differences in postoperative prognosis for HCC patients are large, and these indicators still cannot accurately predict the prognosis of HCC, suggesting that HCC may have different biological subtypes^24,25^.

Dual-phenotype hepatocellular carcinoma (DPHCC) was first reported as a new subtype of HCC in 2011^26^. DPHCC has a typical HCC morphological pattern, but the cancer cells express markers of both HCC and ICC, thus possibly exhibiting dual biological behavior characteristics of HCC and intrahepatic cholangiocarcinoma (ICC)^4,26,27^. The origin of DPHCC is not yet clear, with current research pointing to possible involvement of liver stem cell origin patterns and HCC dedifferentiation patterns, with some reports suggesting that hypoxia can induce evolution from typical HCC to DPHCC^28–30^. At present, the diagnosis of DPHCC relies on the results of pathological immunohistochemistry, with cytokeratin 7 (CK7) and cytokeratin 19 (CK19) being the most commonly used markers^27,31^. Recently, studies have reported that DPHCC, especially the type expressing CK19, has a higher incidence of microvascular invasion, recurrence, and metastasis, and a worse clinical outcome^26,32,33^. Those finding suggest that CK19 plays a crucial role in the invasion and metastasis mechanism of DPHCC. Compared to typical HCC, there is no evidence of significant differences in onset age or HBV infection history among DPHCC patients, but there are significant differences reported in tumor size, AFP expression, and glycoprotein CA199 expression^26,34^.

Computed tomography scan (CT) is a common and important tool for clinical diagnosis of HCC. Due to the neovascularization and abundant arterial blood supply of HCC, the arterial phase enhancement on CT is considered to be an important basis for the diagnosis of HCC^35,36^. However, not all HCC show such typical enhancement features. Some studies reported that HCC with fewer or lacking vascular proliferation showed no obvious enhancement or atypical enhancement features on CT^37,38^. A study reported that the CK19 expression, recurrence rate and mortality in HCC with poor blood supply as demonstrated on enhanced CT images were significantly higher than those in those with rich blood supply^32^. However, the association between different CT enhancement features and the prognosis and pathological markers of HCC has not been widely reported. Therefore, this study aimed to deeply investigate the association among CT imaging, clinical and pathological features of HCC.

## Materials and methods

### Study object

This study retrospectively collected data of liver tumor resection cases performed at the First Affiliated Hospital of Guangxi Medical University from January 1st, 2015 to December 31st, 2015. Inclusion criteria included cases that were pathologically diagnosed with HCC. Exclusion criteria included cases with multiple or metastatic lesions, recurrent cases, cases that had received transcatheter arterial chemoembolization (TACE) before surgery, cases with portal vein tumor thrombus, cases that underwent portal vein ligation before surgery, cases with tumor bleeding or necrosis, cases with indistinct CT lesions, cases with missing preoperative enhanced CT scans, and cases without reported CK7 and CK19 results. This study finally included 97 cases, comprised of 54 cases of typical HCC and 43 cases of DPHCC.

### Clinical data

The clinical medical records and laboratory data were collected retrospectively. All cases were classified as Child–Pugh grade A and BCLC stage A^39^. The preoperative AFP and CA199 were set as a threshold of 400 ng/ml and 37 U/ml respectively^26^. The cut-off for tumor diameter was set at 4 cm, which was the integer closest to the median tumor diameter.

### CT imaging data

All cases underwent plain and contrast-enhanced CT scans of the liver with a slice thickness of 2.0 or 2.5 mm. The arterial, portal vein and delayed phase were scanned at 25, 60 and 120 s after the injection of contrast agent iohexol via an elbow vein, respectively. The average CT values of the liver and tumor at each phase were calculated using the IQQA^®^ liver CT imaging interpretation and analysis system. The CT value of the tumor was the average CT value of the entire three-dimensional spherical tumor, while the CT value of the liver was the average CT value of the liver parenchyma of a sphere with a diameter of approximately 3 cm, avoiding the large vessels. The CT imaging features were evaluated by the ratio of the average CT value of the tumor to the liver (TLR) at each phase.

### Pathological data

The surgical specimens were fixed with formalin (35% to 40% formaldehyde solution) and underwent pathological and immunohistochemical examination by pathologists at the First Affiliated Hospital of Guangxi Medical University, according to the standardized pathological diagnosis guideline for primary liver cancer in China (2015)^27^. HCC biomarkers (GPC-3, HepPar-1 and CD34) and ICC biomarkers (CK7 and CK19) were tested by immunohistochemistry. Briefly, all paraffin slices were subjected to dewaxing and antigen repairing. Endogenous peroxidase activity was blocked using 3% bovine serum albumin. Antibodies against GPC-3, HepPar-1, CD34, CK7, and CK19 were then incubated overnight at 4°C. The following day, the slices were incubated with goat anti-rabbit, followed by staining with 3,3'-diaminobiphenyl chromogen. Finally, the slices were counterstained with hematoxylin. The immunohistochemical results were independently analyzed and reported by two pathologists. The diagnosis of DPHCC was based on the criteria described in the literature: (1) morphological features that match the typical morphological criteria for HCC set by the WHO, including polygonal cancer cells, abundant eosinophilic cytoplasm, cord-like structures resembling liver plates, blood sinuses separating the cord-like structures, and the absence of significant fibrous interstitial tissue^4,27^; (2) immunohistochemical results showing strong positivity in more than 15% of the tumor cells for at least one HCC marker and one ICC marker^26,27^. The presence or absence of nodular formation was used to determine liver cirrhosis.

### Follow-up

The patients underwent outpatient or inpatient re-examination every 3 to 6 months after surgery. As of the last follow-up date, March 20, 2019, 45 cases showed recurrence and 8 cases were lost in recurrence follow-up, with a loss of follow-up rate of 8.2% for recurrence. Meanwhile, 14 cases resulted in death and 20 cases were lost in overall survival follow-up, with a loss of follow-up rate of 20.6% for overall survival (OS).

### Statistical analysis

The association between CT imaging, clinical, and pathological features was analyzed using the *t*-test and chi-square test. Univariate recurrence-free survival (RFS) and OS analysis was performed using the log-rank test. Multivariate RFS analysis was performed using the Cox proportional hazard model. All statistical analysis was performed using R (version: 3.5.3). *P* value of less than 0.05 was regarded as statistically significant.

### Ethics approval and consent to participate

This study was conducted in accordance to the Declaration of Helsinki and approved by the Ethics Committee of the First Affiliated Hospital of Guangxi Medical University. Due to the retrospective nature of the study, the need for informed consent was waived by the Ethics Committee of the First Affiliated Hospital of Guangxi Medical University.

## Results

### Correlation analysis of clinical and pathological features

The study involved 97 cases of HCC, consisting of 83 males and 14 females, with a median age of 47 years (25–76 years). The positive rates of serum hepatitis B virus surface antigen (HBsAg), CK7 and CK19 were 84.5%, 35.1% and 20.6% respectively. The positive rate of CK19 was significantly higher in cases with age < 47 years (*P* = 0.005), tumor diameter > 4 cm (*P* = 0.016) or AFP ≥ 400 ng/ml (*P* = 0.007). However, no significant association was found between pathological subtypes or the expression of CK7 with gender, nationality, age, body mass index (BMI), tumor diameter, AFP, CA199, HBsAg and liver cirrhosis (all *P* > 0.05) (Table 1).

**Table 1:** The results of correlation analysis of clinical and pathological features.

Clinical features		Subtype			CK7			CK19		
		THCC	DPHCC	*P*	( −)	( +)	*P*	( −)	( +)	*P*
Gender	Male	49	34	0.182	53	30	0.805	69	14	0.062
	Female	5	9		10	4		8	6	
Nationality	Han	33	21	0.316	38	16	0.298	43	11	1.000
	Zhuang	21	22		25	18		34	9	
Age	< 47 years	24	24	0.364	31	17	1.000	32	16	0.005**
	≥ 47 years	30	19		32	17		45	4	
BMI	< 24	38	32	0.831	46	24	0.986	53	17	0.247
	≥ 24	16	11		17	10		24	3	
Diameter	≤ 4 cm	28	17	0.316	28	17	0.756	41	4	0.016*
	> 4 cm	26	26		35	17		36	16	
AFP	< 400 ng/ml	35	22	0.250	36	21	0.822	51	6	0.007**
	≥ 400 ng/ml	19	21		27	13		26	14	
CA199	< 37 U/ml	45	35	1.000	52	28	1.000	63	17	0.997
	≥ 37 U/ml	9	8		11	6		14	3	
HBsAg	(-)	8	7	1.000	10	5	1.000	12	3	1.000
	( +)	46	36		53	29		65	17	
Cirrhosis	No	21	13	0.501	23	11	0.852	27	7	1.000
	Yes	33	30		40	23		50	13	

*THCC* typical hepatocellular carcinoma, *DPHCC* dual-phenotype hepatocellular carcinoma, *BMI* body mass index, *HBsAg* hepatitis B virus surface antigen.

*P < 0.05; **P < 0.01.

### Correlation analysis of imaging and pathological features

**Figure 1:**
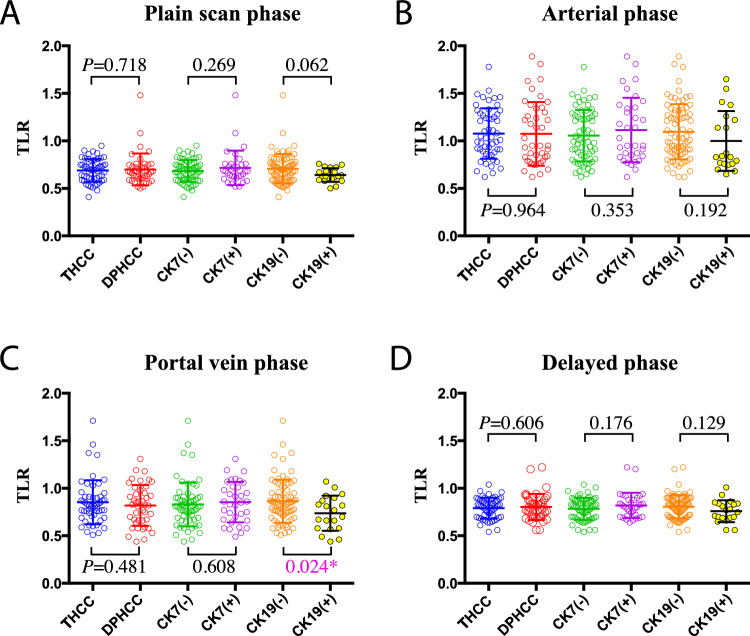
Comparison of TLR in each phase of pathological features. *TLR* the average CT value of the tumor to the liver, *THCC* typical hepatocellular carcinoma, *DPHCC* dual-phenotype HCC.

**Figure 2:**
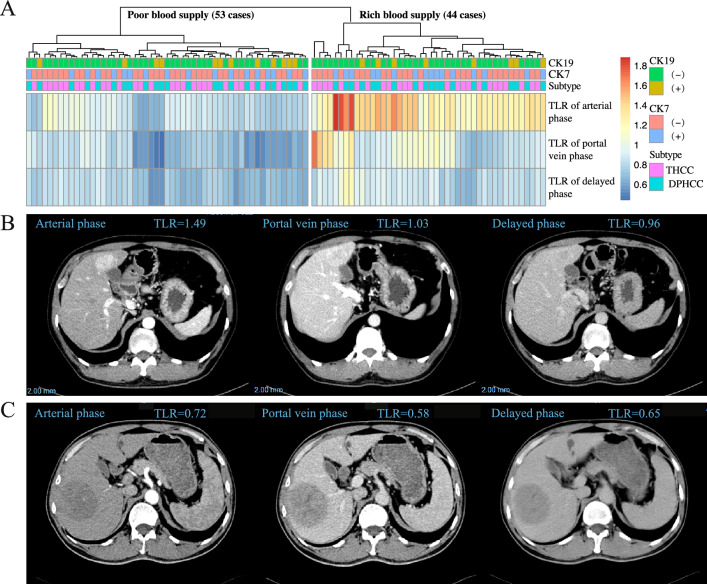
The TLR cluster heat map and typical imaging. (**A**) TLR cluster heat map for TLR of the arterial, portal vein and delayed phases; (**B**) Typical imaging of rich blood supply (TLR high) case; (**C**) Typical imaging of poor blood supply (TLR low) case. *TLR* the average CT value of the tumor to the liver, *THCC* typical hepatocellular carcinoma, *DPHCC* dual-phenotype HCC.

The TLR of CK19 positive group is significantly lower than CK19 negative group in portal vein phase (*P* = 0.024), but no significant correlation was found in the plain scan, arterial and delayed phase (all *P* > 0.05). There were no significant differences in TLR between different pathological subtypes and CK7 expression groups in each phase (all *P* > 0.05) (Fig. 1). Clustering analysis was performed on the TLR of the arterial, portal vein and delayed phases and the result showed that the cases mainly divided into two groups: one group is the rich blood supply type with obvious enhancement in arterial phase and relative decline in the portal vein and delayed phase (44 cases); the other group is the poor blood supply type with no obvious enhancement in the arterial, portal vein and delayed phase (53 cases) (Fig. 2A). The typical imaging of rich blood supply (TLR high) and poor blood supply (TLR low) cases were showed in Fig. 2B and C respectively. Correlation analysis was performed on the above clustering groups and the pathological features, but no significant correlation was found (all *P* > 0.05) (Table 2).

**Table 2:** The results of correlation analysis of imaging and pathological features.

CT imaging features	Subtype			CK7			CK19		
	THCC	DPHCC	*P*	( −)	( +)	*P*	( −)	( +)	*P*
Poor blood supply	29	24	0.998	35	18	0.974	41	12	0.773
Rich blood supply	25	19		28	16		36	8	

*THCC* typical hepatocellular carcinoma, *DPHCC* dual-phenotype HCC.

### Correlation analysis of clinical and imaging features

Correlation analysis was performed on the above clustering groups and the clinical features and the result indicated that a significant correlation was found between the rich or poor blood supply type and tumor diameter (*P* = 0.013), while no significant correlation was found with patient gender, nationality, age, BMI, AFP, CA199, HBsAg and liver cirrhosis (all *P* > 0.05) (Table 3).

**Table 3:** The results of correlation analysis of clinical and imaging features.

Clinical features		CT imaging features		
		Poor blood supply	Rich blood supply	*P*
Gender	Male	45	38	1.000
	Female	8	6	
Nationality	Han	28	26	0.680
	Zhuang	25	18	
Age	< 47 years	28	20	0.604
	≥ 47 years	25	24	
BMI	< 24	37	33	0.734
	≥ 24	16	11	
Diameter	≤ 4 cm	18	27	0.013*
	> 4 cm	35	17	
AFP	< 400 ng/ml	26	31	0.054
	≥ 400 ng/ml	27	13	
CA199	< 37 U/ml	47	33	0.135
	≥ 37 U/ml	6	11	
HBsAg	(−)	7	8	0.695
	( +)	46	36	
Cirrhosis	No	17	17	0.645
	Yes	36	27	

*BMI* body mass index, *AFP* alpha-fetoprotein, *HBsAg* hepatitis B virus surface antigen.

*P < 0.05.

### Association of clinical features and clinical outcome

The median follow-up time for recurrence was 38 months (1–50 months). The univariate RFS analysis showed that cases with tumor diameter > 4 cm (HR: 2.05, 95% CI 1.11 to 3.78, *P* = 0.019), AFP ≥ 400 ng/ml (HR: 2.50, 95% CI 1.37 to 4.54, *P* = 0.002) or CA199 ≥ 37 U/ml (HR: 2.23, 95% CI 1.12 to 4.42, *P* = 0.020) had a higher recurrence rate, while there was no significant correlation between patient gender, nationality, age, BMI, HBsAg, liver cirrhosis or postoperative adjuvant transcatheter arterial chemoembolization (TACE) and HCC RFS (all *P* > 0.05) (Table 4). The univariate OS analysis showed that cases with AFP ≥ 400 ng/ml (HR: 3.68, 95% CI 1.15 to 11.73, *P* = 0.019) had a worse OS, while there was no significant correlation between patient gender, nationality, age, BMI, tumor diameter, CA199, HBsAg, liver cirrhosis or postoperative adjuvant TACE and HCC OS (all *P* > 0.05) (Table 5).

**Table 4:** The results of univariate RFS analysis of clinical features.

Clinical features		N	Recurrence	MRT (month)	HR (95%CI)	*P*
Gender	Male	81	39	44	1.00	
	Female	14	6	NA	0.78 (0.33, 1.85)	0.573
Nationality	Han	52	22	NA	1.00	
	Zhuang	43	23	31	1.69 (0.94, 3.05)	0.078
Age	< 47 years	48	24	45	1.00	
	≥ 47 years	47	21	NA	0.90 (0.50, 1.62)	0.737
BMI	< 24	68	34	44	1.00	
	≥ 24	27	11	NA	0.69 (0.35, 1.36)	0.279
Diameter	≤ 4 cm	45	16	NA	1.00	
	> 4 cm	50	29	18	2.05 (1.11, 3.78)	0.019*
AFP	< 400 ng/ml	55	18	NA	1.00	
	≥ 400 ng/ml	40	27	18	2.50 (1.37, 4.54)	0.002**
CA199	< 37 U/ml	79	34	NA	1.00	
	≥ 37 U/ml	16	11	15	2.23 (1.12, 4.42)	0.020*
HBsAg	( −)	15	6	NA	1.00	
	( +)	80	39	45	1.34 (0.57, 3.16)	0.507
Cirrhosis	No	33	13	NA	1.00	
	Yes	62	32	41	1.32 (0.69, 2.52)	0.394
TACE	No	58	29	44	1.00	
	Yes	37	16	NA	0.75 (0.41, 1.39)	0.370

*MRT* median recurrence time, *HR* hazard ratio, *CI* confidence interval, *BMI* body mass index, *AFP* alpha-fetoprotein, *HBsAg* hepatitis B virus surface antigen, *TACE* transcatheter arterial chemoembolization.

*P < 0.05; **P < 0.01.

**Table 5:** The results of univariate OS analysis of clinical features.

Clinical features		N	Dead	MST (month)	HR (95%CI)	*P*
Gender	Male	81	12	NA	1.00	
	Female	14	2	NA	0.91 (0.20, 4.05)	0.896
Nationality	Han	52	7	NA	1.00	
	Zhuang	43	7	NA	1.49 (0.52, 4.25)	0.450
Age	< 47 years	48	9	NA	1.00	
	≥ 47 years	47	5	NA	0.53 (0.18, 1.57)	0.244
BMI	< 24	68	11	NA	1.00	
	≥ 24	27	3	NA	0.59 (0.17, 2.13)	0.419
Diameter	≤ 4 cm	45	4	NA	1.00	
	> 4 cm	50	10	NA	2.79 (0.87, 8.9)	0.072
AFP	< 400 ng/ml	55	4	NA	1.00	
	≥ 400 ng/ml	40	10	NA	3.68 (1.15, 11.73)	0.019*
CA199	< 37 U/ml	79	12	NA	1.00	
	≥ 37 U/ml	16	2	NA	0.95 (0.21, 4.25)	0.951
HBsAg	(-)	15	1	NA	1.00	
	( +)	80	13	NA	2.36 (0.31, 18.09)	0.391
Cirrhosis	No	33	5	NA	1.00	
	Yes	62	9	NA	0.89 (0.30, 2.67)	0.840
TACE	No	58	11	NA	1.00	
	Yes	37	3	NA	0.37 (0.10, 1.34)	0.116

*MST* median survival time, *HR* hazard ratio, *CI* confidence interval, *BMI* body mass index, *AFP* alpha-fetoprotein, *HBsAg* hepatitis B virus surface antigen, *TACE* transcatheter arterial chemoembolization.

*P < 0.05.

### Association of pathological features and clinical outcome

**Figure 3:**
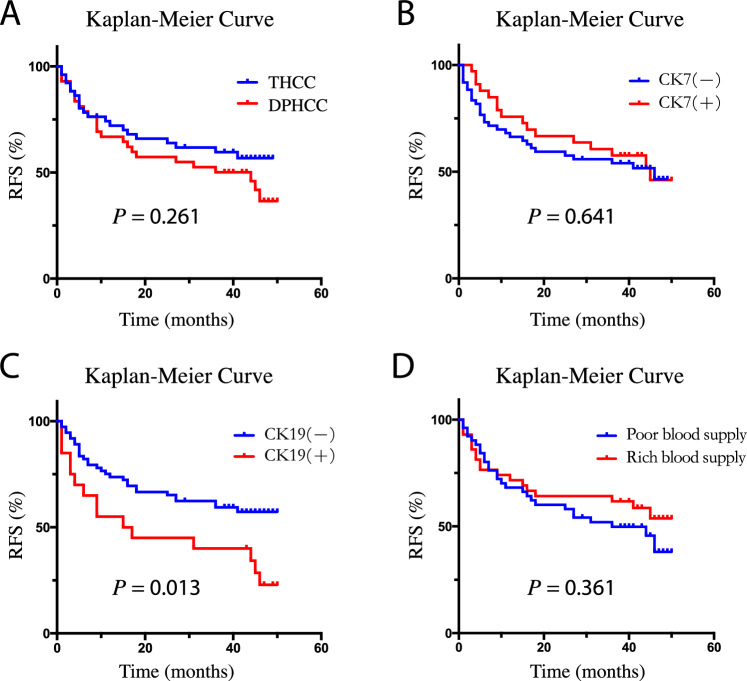
The RFS curve of imaging and pathological features. (**A**) RFS curve of pathological subtypes; (**B**) RFS curve of CK7 positive or negative group; (**C**) RFS curve of CK19 positive or negative group; (**D**) RFS curve of imaging features. *RFS* recurrence-free survival, *THCC* typical hepatocellular carcinoma, *DPHCC* dual-phenotype HCC.

**Figure 4:**
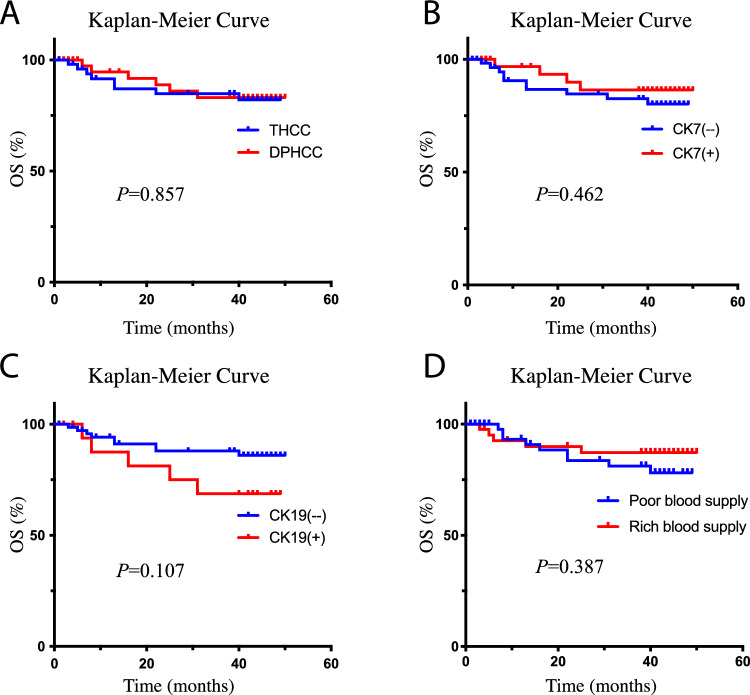
The OS curve of imaging and pathological features. (**A**) OS curve of pathological subtypes; (**B**) OS curve of CK7 positive or negative group; (**C**) OS curve of CK19 positive or negative group; (**D**) OS curve of imaging features. *OS* overall survival, *THCC* typical hepatocellular carcinoma, *DPHCC* dual-phenotype HCC.

The univariate RFS analysis of pathological features showed that cases with CK19 positive expression had a higher recurrence rate than CK19 negative expression (HR, 2.17; 95% CI, 1.16–4.04; *P* = 0.013), while there was no significant correlation between the overall pathological subtype or CK7 expression and RFS (all *P* > 0.05). For multivariate analysis, pathological subtype, CK7 and CK19 expression were respectively included into a Cox proportional hazard model with the tumor diameter, AFP and CA199, which significantly related to RFS, but the results showed that pathological subtype, CK7 or CK19 expression were not significantly related to HCC RFS (all *P* > 0.05) (Fig. 3A–C, Table 6). Pathological subtype, CK7 or CK19 expression were not significantly related to HCC OS in the univariate and multivariate analysis (all *P* > 0.05) (Fig. 4A–C, Table 7).

**Table 6:** The results of RFS analysis of imaging and pathological features.

Pathological features	N	Recurrence	MRT (month)	Univariate analysis		Multivariate analysis	
				HR (95% CI)	*P*	HR (95% CI)	*P*
Subtype							
THCC	52	21	NA	1.00		1.00	
DPHCC	43	24	44	1.40 (0.78, 2.51)	0.261	1.12 (0.61, 2.06)	0.705
CK7							
( −)	61	29	46	1.00		1.00	
( +)	34	16	45	0.86 (0.47, 1.59)	0.641	0.94 (0.51, 1.74)	0.839
CK19							
( −)	75	30	NA	1.00		1.00	
( +)	20	15	16	2.17 (1.16, 4.04)	0.013*	1.46 (0.74, 2.88)	0.276
CT imaging features							
Poor blood supply	52	27	36	1.00		1.00	
Rich blood supply	43	18	NA	0.76 (0.42, 1.38)	0.361	0.98 (0.51, 1.89)	0.948

*MRT* median recurrence time, *HR* hazard ratio, *CI* confidence interval, *THCC* typical hepatocellular carcinoma, *DPHCC* dual-phenotype HCC.

*P < 0.05.

**Table 7:** The results of OS analysis of imaging and pathological features.

Pathological features	N	Dead	MST (month)	Univariate analysis		Multivariate analysis	
				HR (95%CI)	*P*	HR (95%CI)	*P*
Subtype							
THCC	52	8	NA	1.00		1.00	
DPHCC	43	6	NA	0.91 (0.31, 2.61)	0.857	0.77 (0.27, 2.23)	0.631
CK7							
( −)	61	10	NA	1.00		1.00	
( +)	34	4	NA	0.65 (0.2, 2.07)	0.462	0.69 (0.22, 2.2)	0.532
CK19							
( −)	75	9	NA	1.00		1.00	
( +)	20	5	NA	2.38 (0.8, 7.11)	0.107	1.59 (0.51, 4.99)	0.428
CT imaging features							
Poor blood supply	52	9	NA	1.00		1.00	
Rich blood supply	43	5	NA	0.62 (0.21, 1.85)	0.387	0.86 (0.28, 2.67)	0.799

*MST* median survival time, *HR* hazard ratio, *CI* confidence interval, *THCC* typical hepatocellular carcinoma, *DPHCC* dual-phenotype HCC.

### Association of imaging features and clinical outcome

The univariate RFS analysis of imaging features showed that the rich or poor blood supply type was not significantly related to HCC RFS (*P* = 0.361). For multivariate analysis, the rich or poor blood supply type was included into a Cox proportional hazard model with the tumor diameter, AFP and CA199, which significantly related to RFS, but the results showed that the rich or poor blood supply type was still not significantly related to HCC RFS (*P* = 0.948) (Fig. 3D, Table 6). The rich or poor blood supply type was not significantly related to HCC OS in the univariate and multivariate analysis (all *P* > 0.05) (Fig. 4D, Table 7).

## Discussion

Hepatocytes, bile duct epithelial cells and hepatoma cells all contain cytoskeletal intermediate filaments. However, different types of cancer cells have different characteristic combinations of CK proteins. Hepatocytes and typical hepatocellular carcinoma cells usually express CK8 and CK18, while bile duct cells and their malignant cholangiocarcinoma cells usually express CK7 and CK19^40,41^. Although both CK7 and CK19 are expressed in bile duct cells, CK7 is also considered as a marker of intermediate hepatocytes, while CK19 is considered as a marker of Hepatic progenitor cells (HPC) and bile duct differentiation^28^.

In recent years, there have been reports that CK7 and CK19 have also been proved to be expressed in some HCC^34,42^. This type of HCC was called DPHCC, a higher malignant subtype^26^. Many studies have shown that the CK19 positive expression is related to the early recurrence of HCC after hepatectomy, radiofrequency ablation or liver transplantation^32,42–44^. Therefore, CK19 positive expression is considered a poor prognostic factor for HCC^45^. This study also observed a similar result, which among the 97 HCC cases with BCLC stage A included in this study, the risk of recurrence after liver tumor resection was significantly increased in cases with CK19 positive expression. However, the mechanism of how CK19 affects the prognosis of HCC is currently not clear. Study by Govaere et al. showed that expression of CK19 is closely related to tumor size (also observed in this study), tumor differentiation, metastasis, and microvascular invasion. Further microarray and microRNA analysis showed that CK19 positive HCC highly expressed invasion or metastasis related markers, bile duct or HPC markers and microRNA family 200 members (such as miR-141, miR-200c)^33^. In addition, study by Van et al. showed that compared with CK19 negative hepatocellular adenoma, CK19 positive HCC significantly expressed higher glypican-3, B-cell specific murine leukemia virus integration site 1, adhesion proteins, integrin beta-1/CD29, and prominin-1/CD133^46^. This expression pattern suggests that CK19 positive HCC is higher invasive.

In the current study, we also observed a higher expression rate of CK19 in young-onset hepatocellular carcinoma (HCC) (< 47 years, 33.3% vs. 8.2%, P = 0.005). Au KY et al. similarly reported a significantly higher expression of CK19 in HCC patients below 40 years of age (61%) compared to results from other studies (10–30%)^47^. Klein et al. also demonstrated a notable increase in CK19 expression in HCC patients under 30 years of age (22%)^48^. The enhanced expression of CK19 in young-onset HCC may indicate an increased stemness signature in this subset of patients, suggesting a higher malignancy and poorer prognosis for young-onset HCC. However, the mechanisms underlying the higher expression of CK19 in young-onset HCC have not been elucidated, and further research may be needed to clarify this aspect in the future.

As mentioned above, DPHCC, especially the type of CK19 positive expression, has higher invasiveness, risk of metastasis and recurrence. Therefore, if the diagnosis of DPHCC can be predicted according to preoperative examination, it may provide important basis for clinical individualized treatment. However, at present, the diagnosis of DPHCC mainly depends on the pathological and immunohistochemical results after surgery^27^. Compared with the clinical features of typical HCC, there is no evidence that there are significant differences in nationality and sex, obesity, cirrhosis, HBV or HCV infection history among DPHCC, but there are significant differences in tumor size, AFP and CA199 expression reported in the previous study^26,33^. This study also observed a similar result, which among the 97 HCC cases with BCLC stage A included in this study, the age, tumor size and AFP expression of patients were significantly correlated with the expression of CK19. In addition, enhanced CT is an important tool for the clinical diagnosis of HCC. Study by Chung et al. showed that the frequency, recurrence rate and mortality of CK19 expression in HCC with poor blood supply on enhanced CT sequence images were significantly higher than those of HCC with rich blood supply^32^. However, this study only observed a significant difference between the TLR value and CK19 expression in the portal vein phase, while no significant difference was found in the overall enhancement characteristics of the three phases and the RFS of HCC.

In addition, some limitations in this study need to be pointed out. Firstly, as a single-center study, the results should be confirmed by larger, multi-center studies. Additionally, our initial study design was primarily focused on observing RFS, hence the minimum follow-up duration was set at about 3 years and the maximum observation period at about 4 years. This duration may be insufficient for observing OS for BCLC stage A HCC. At the same time, the follow-up in this study had a certain rate of loss to follow-up, which may introduce some bias to the research results.

## Conclusion

In summary, the results of this study suggest that CK19 expression may be associated with the enhancement feature of the portal vein phase CT image, and CK19 positive may suggest a worse RFS. However, replication of these findings in larger, multi-center studies is needed.

## Data Availability

All data used by or generated in this study is available from the corresponding author upon reasonable request.

## Abbreviations

DPHCC: Dual-phenotype hepatocellular carcinoma
HCC: Hepatocellular carcinoma
CT: Computerized tomography scan
TLR: The ratio of the average CT value of tumor to liver
RFS: Recurrence-free survival
CK7: Cytokeratin 7
CK19: Cytokeratin 19
BCLC: Barcelona clinical liver cancer staging
AFP: Alpha-fetoprotein
PVTT: Portal vein tumor thrombus
ICC: Intrahepatic cholangiocarcinoma
TACE: Transcatheter arterial chemoembolization
HBsAg: Hepatitis B virus surface antigen
BMI: Body mass index
HPC: Hepatic progenitor cells
